# The challenges with the validation of research antibodies

**DOI:** 10.12688/f1000research.10851.1

**Published:** 2017-02-17

**Authors:** Jan L.A. Voskuil

**Affiliations:** 1 Aeonian Biotech, Marcham, OX13 6PH, UK

**Keywords:** Antibodies, validation, reproducibility, conformance

## Abstract

This article further discusses the reproducibility crisis in biomedical science and how poor conduct of commercial antibodies contribute to this. In addition, the way quality data are presented on product sheets by antibody vendors is scrutinized. The article proposes that there is a distinction between testing data and validation data, and special attention is asked for consistency between batches and aliquots. Moreover, the article separates the specifics, such as formulation, antigen and price, from the specifics on performance. Finally, a two-tier approach is discussed, enabling scientists to anticipate how an antibody is likely to perform when repeated purchases are required.

## Introduction

In the scientific community, there is growing attention to the quality of commercial research antibodies, particularly since the recent intensified publications on the crisis of reproducibility ^1– 6^. Although some papers have already addressed the lack of quality in the antibody market much earlier ^7– 10^, since a link was made between lack of scientific reproducibility and antibody conduct ^11– 13^, more efforts were made to bring all stakeholders in the research antibody market together to move forward. Such efforts resulted in online discussions ( https://www.protocols.io/groups/gbsi-antibody-validation-online-group), publications on validation ^14– 17^ and two international meetings ^18– 20^. Everyone agreed that to some extent bad quality antibodies may contribute to lack of scientific progress and that something had to be done to remove such blame from the industries. The strong message is that antibodies need proper validation first before being used in scientific research. A few large vendors have commenced with exhaustive validation for some of their products, but the investment for validation of each individual product is very high and such efforts are not commercially attractive enough to apply for all catalogue items when the size of the catalogue is in the hundreds of thousands ^21^. Besides, despite all the good intensions and large investments in the industry, the approach of exhaustive validation is not the complete answer to the problem. When it comes to antibody validation there are some practical difficulties that are not always appreciated, or they are underestimated if not totally ignored. This article aims to create clarity in the practical issues that directly affects the quality and performance of research antibodies, even when a product has successfully gone through an exhaustive validation process.

## Basic principles of validation

There is a fundamental difference between testing an antibody in a certain application, and validation. The former is put in practice by most of us (both vendors/manufacturers and research scientists). Until recently testing with a positive result was more than adequate to pass a product for the market and to persuade researchers to buy the tested antibody. For example, when an antibody was tested in Immunohistochemistry (IHC) and there was a signal, the vendor would go ahead adding the data to the product sheet and adding IHC to the tested applications. Any scientist would not think otherwise than to assume this antibody was fit for IHC and to buy the product, especially when the brand is large and deemed reliable. These times are over. Currently a signal needs to be in the right place and in a relevant tissue to be credible.

Validation goes way beyond mere testing. Here, we first consider how the antibody is commonly used. For example, a CD4 antibody is most likely being used in Flow Cytometry (FC). Then it follows that this antibody is primarily tested in FC and not in Western Blot (WB) or IHC. However, for proper validation the signal needs to be specific and selective; that is at the maximal dilution for good signal in the right cell type, there should be hardly any signal in the wrong cell types. Hence, validation always involves comparison between expressing and non-expressing cells or tissues at identical antibody dilutions. A CD4 antibody is validated in FC when it lifts out a proportionate sub population from all T-cells (the proportion of CD4+ T cells). The way to do this is to have all T cells selected from the buffy coat first by a generic T cell marker antibody (formerly and fully validated for this purpose) and have the signal of CD4 label related to the total T cell signal quantified. Ideally there is another validated CD4 antibody to compare with and to confirm that the observed proportion of CD4 signal relative to the total T cell signal is consistent across the two CD4 antibodies. A commonly used format showing a stain distribution of a single cell line with a peak away from background is not evidence of specificity. For IHC or WB, again comparison between expressing and non-expressing cells/tissues is required for proper validation. An antibody fit for and validated in WB will not automatically pass in IHC or FC though. The notion in the literature ^7^ that every antibody needs first validation in WB before moving on to the required assay is flawed and entails the risk of losing out on precious FC antibodies that will never work in WB or IHC.

## Conformity of validated antibodies in batches and aliquots

In an ideal world, all antibodies on offer are fully validated for the applications on demand by the market. Although we are far away from this reality, all vendors and manufacturers are currently working very hard to reach this goal. Consequently, increasing amounts of fully validated products are emerging daily. However, this is not the end of the tragedy. As discussed thoroughly in multimedia and to a smaller extent in the literature ^8, 14^, the antibodies on sale come in batches or lots. And there will be variabilities from batch to batch or from lot to lot. This is true for monoclonal antibodies (especially when sold in an undefined formulation, such as culture media or ascites), but to a much larger extent this is the case for polyclonal antibodies (especially for undefined formulations, such as serum or plasma, but also for antibodies raised to the entire protein and with an undefined epitope). Therefore, the test/validation results shown on the product sheet will no longer be representative after the batch or lot has been replaced by its successor, unless the data have been reproduced with the new batch/lot.

There is confusion about the terms batch and lot. They are generally used interchangeably. There is a strong case though to distinguish batches from aliquots: It is recommended to have a batch defined by the harvest and purification, while an aliquot is defined by the place and the day a stock vial is split. The term lot is best avoided to keep the separation between batch and aliquot unambiguous. This article proposes to have this principle copied worldwide. The functionality of this distinction is that any non-conformity can be easily traced back either to inactivation by storage or transit (then a different aliquot with a different history will show conformity again), or to a bad purification or bad production (in which case the entire batch will be withdrawn from the market and be replaced by a new batch).

It is recommended to have transparency regarding batches and aliquots. The batch codes are preferred to be visible on the product sheet, while both the batch code and aliquot coding is required to be specified on the label of every vial.

## Responsibility of testing and validation

As soon as a purchased antibody has arrived, it is the responsibility of the scientist to make sure the product arrived in proper conditions. It would be good practice to start reproducing the data as described on the product sheet to make sure the antibody shows conformity. This should be done before splitting the product into aliquots and storage in a (non-cycling) freezer. This way a non-conforming product can be returned or the specifics on the label can be forwarded to the vendor together with the complaint. Any self-respecting vendor will either replace or refund when a product is non-conformant. Once the antibody has demonstrated its integrity, it is time to use it in the intended experiments. No matter the high quality of data shown on the product sheet, every scientist must validate the antibody in the assay and biological material of interest. It is not evident at all that a positively tested antibody on liver or kidney is going to work on fibroblast or neuronal cell lines. In addition, one should not assume that positive result on a lysate of a neuroblast cell line in WB means that the scientist is going to get the antibody to work in lysates of different brain regions. So, the scientist is primarily responsible for the validation of the purchased antibody in the very defined conditions of the experiments to be done. A lot of precious time and biological research material is saved by following the above steps before using the purchased antibody for the intended experiments. Most vendors and manufacturers will most likely not go much further than confirmation of their products in one or a few assay types in one or a few cell types. Vendor and scientist will achieve a shared responsibility when they develop a mutual understanding and respect for each other’s objectives ^21^.

## Deciding factors on the product of choice

Given the size and complexity of the research antibody market, the best way to decide which antibody to pick is to consider a two-tier approach. The first tier considers the specifications of the product regardless of its performance (see Table 1). The scientist needs to decide if a mono-specific antibody is required (which may be essential for certain assays when dependent on repeat purchases), and how the product is formulated. These considerations need to be weighed against the clone/batch specifications, presence of quality data and price. The second tier considers the claimed performance, as specified on the product sheet. Here, the scientific integrity of the quality data come into play (see Table 2). There is an important distinction to be made by the scientist if the antibody is required for native conditions or for non-native conditions. Antibodies confirmed in native assays may not work in non-native assays and vice versa. The extent of quality data, as described on the product sheet, is incrementally listed for each of the most common assay types.

**Table 1.** Tier 1: Performance-independent specifications. Overview of variety on performance-independent specification visible on the vendor’s product sheet. WB: Western Blot.

Antigen	Clone/batch specification	Quality data to back up application codes	Formulation	Quantity/price
Entire protein/large part of protein	Nothing specified	No data and no literature references	Serum, culture supernatant, ascitus	No quantity, ask for quotation
Epitope/short peptide sequence, undisclosed	Clone # specified	No data, but proven record through literature	IgG-fraction	Good for # WBs
Epitope/short peptide sequence, disclosed	Batch available on request	Data not batch-specific	Affinity purified without specification	Volume
	Batch on product sheet/cat #-spec	Data batch-specific but not specified	Protein A or G purified	Specified IgG in ug or mg
		Data batch-specific and specified	Antigen affinity purified	

**Table 2.** Tier 2: Performance specifications. Overview of variety of performance specifications visible on the vendor’s product sheet. NB: Comparison between wildtype and knock-out is in all cases the best validation and is not incorporated in this schedule. Cell type, a cell line or a cell type from primary culture or a cell type within a mixture of types/tissue; KD: Knock-Down by induced siRNA expression; RT-PCR, quantitative data demonstrated the levels of mRNA in KD relative to wildtype levels; WB: Western Blot; IP: Immunoprecipitation.

*Native conditions*
**ELISA**
On coated purified peptide On coated purified entire protein Sandwich in buffer with purified protein for calibration Sandwich in natural matrix, spiked with purified protein for calibration Sandwich in natural matrix comparing naturally low and high level samples
**Flow Cytometry**
Irrelevant cell type, one ab only and without any controls Irrelevant cell type, one ab only and with isotype control Relevant cell type, one Ab only and without any controls Relevant cell type, one Ab only and with isotype control Relevant cell type mixed with a non-expressing cell type and co-stained with a sharing marker Mixed cell types with a subpopulation stained by a shared marker, and part of it stained by research antibody
**Immunocytochemistry**
Irrelevant cell type without comparisons Irrelevant cell type compared with KD but without RT-PCR data Irrelevant cell type compared with KD and with RT-PCR data Relevant cell type without comparisons Relevant cell type compared with KD but without RT-PCR data Relevant cell type compared with KD and with RT-PCR data
*Non-native conditions*
**Western Blot**
WB on overexpression only WB on irrelevant cell type at endogenous levels WB on relevant cell type but wrong band(s) WB on relevant cell type with correct band(s), but no further controls WB on relevant cell type with correct band(s), and with controls:
**Immunoprecipitation**
IP without proper controls, detected by WB with same antibody IP without proper controls, detected by WB with a different antibody IP without proper controls, detected directly through radiogram or fluorescence IP comparing with and without primary, detected by WB with same antibody IP comparing with isotype, detected by WB with same antibody (using light chain or heavy chain detected by secondary as loading control) IP comparing with and without primary, detected by WB with a different antibody IP comparing with isotype, detected by WB with a different antibody (not having loading control) IP comparing with isotype, detected by WB with a different antibody (but having loading control by other means)
**Immunohistochemistry**
Unclear cellular location staining on cancerous tissue only Irrelevant tissues and with unclear cellular location staining Irrelevant tissues but with clear and correct cellular location staining Relevant tissues, but unclear cellular location staining Relevant tissues, with clear cellular location staining

The two tables highlight a sliding scale of quality specifications currently offered on many catalogues worldwide. We should not dismiss vendors and manufacturers for not having the highest level of quality specifications available for each single product because of the practical restrictions coming with the size and resources of every company ^21^. It is down to the scientist to find their way, and in the meanwhile the manufacturers and vendors do their utmost to deserve the scientist’s trust in their quality. Nonetheless, Table 2 demonstrates that many product sheets show inadequate information and are not yet meeting current requirements in the market. There will be increasing demand for testing in biological relevant cell types/tissues or when gene expression allows to have comparative data to validate the observed signals against negative controls.

In addition, product sheets of many peptide-generated antibodies show an ELISA titre to the immunizing peptide, but they usually claim ELISA in the tested application list, which is deceiving because this claim is read as any type of ELISA involving detection of entire protein. When the antibody was merely tested on peptide-coated micro-wells, it would be better to claim peptide-ELISA as the tested application rather than ELISA. We do see more often the application code IHC better specified as IHC-p (paraffin-embedded) and IHC-fr (frozen sections). Similarly, we could use ELISA-p (peptide or protein coated wells) and ELISA-s (sandwich).

## Reproducibility and specificity

Any proper validation must include evidence of robustness from batch to batch. External factors, such as exposure to freeze/thaw cycles, and to radiation or extreme heat, will affect the integrity of the antibody. An inactivated aliquot may show either lack of signal, or non-specific signal. Batch variations are subject to variations from animal to animal and from purification to purification. It is worth mentioning that undefined formulations, as described in Table 1 column 4, will have a profound effect on the reproducibility from batch to batch and needs serious consideration especially by assay/kit developers who depend on long term supply of product with identical characteristics from order to order. Antibodies with a defined epitope/immunizing peptide are intrinsically more robust compared to antibodies raised to entire proteins because the limited size of the antigen increases the chance of reproducible characteristics ^8^. This principle can only be overruled when large amounts of animals are immunized with the same entire protein and their antibodies are pooled together to reach a gold standard. However, potential cross-reactivity to related other proteins needs to be considered as well. This is not possible for monoclonal antibodies without known epitope mapping, and in such cases validation must include testing of cross-reactivity directly to such related proteins.

## Discussion

The considerations set out above can be used as a starting point to generate scoring systems. Many vendors are already doing this. However, research scientists remain unaware of such scoring as they are used for internal purposes only. Although such practice will ultimately lead to a much higher quality product on the market, for the moment there is a need for research scientists and assay developers to find their way when looking for that specific antibody fit for their special set-up. Up to this point, they are reliant on cited literature and the reputation of the vendor. However, because of exchange of products across catalogues ^8, 20^, a situation is created that it is no longer evident from the product sheet if the antibody is offered by the original manufacturer and if the associated quality data is still representative for the current batch on sale. In addition, each large catalogue has several antibodies to the same protein. This makes the choice for the scientist difficult, especially when the cited literature does not specify the catalogue number, and the manufacturer will not be able to tell which one of their products was used for the experiments shown in that paper. This omission has been recognized and publishers are no longer expected to accept a paper without the catalogue numbers of the antibodies used. Therefore, any guidance industries can provide to facilitate biomedical research in finding the right antibody for the specific needs would be more than welcome. In the meanwhile, one is dependent on advice from individual insiders of the industries as they know all relevant details that may not be visible by the public. Such advisers will be best equipped to sift out the best candidate antibodies from the different catalogues for initial testing, followed by proper validation.
