# Supplementary material for: The challenges with the validation of research antibodies
Source: F1000Res. 2017 Feb 17;6:161. [Version 1] doi: 10.12688/f1000research.10851.1 (PMC5333605; doi:10.12688/f1000research.10851.1)
Supplement: Peer review history

# Peer review: Referee response for version 1

**DOI:** 10.5256/f1000research.11700.r20350

**Published:** 2017-03-16

**Authors:** Simon Glerup

**Affiliations:** 1 Department of Biomedicine, Aarhus University, Aarhus, Denmark

This opinion article makes an important contribution to the ongoing and growing discussion of the use of antibodies in research. The main point is that validation goes beyond mere testing and that when selecting an antibody it is of critical importance for scientists to consider the data provided by the manufacturer in the light of the actual experiment it is intended for. This includes considerations of the relevant tissue, cell type and technique as well as the use of proper positive and negative controls.

 Minor comments:

1. I strongly agree with Referee 2 regarding clarification of the statement ”Antibodies with a defined epitope / immunizing peptide are intrinsically more robust compared to antibodies raised to the entire proteins because the limited size of the antigen increases the chance of reproducible characteristics”. Such statement needs to be accompanied by references to actual data showing this. The current reference 8 is a review.
2. I consider the sentence “and in the meanwhile the manufacturers and vendors do their utmost to deserve the scientist’s trust in their quality” highly subjective. This may be true in certain cases but I do not think that this can be extended to describe the behavior of the entire industry.

I have read this submission. I believe that I have an appropriate level of expertise to confirm that it is of an acceptable scientific standard.

# Peer review: Referee response for version 1

**DOI:** 10.5256/f1000research.11700.r20349

**Published:** 2017-03-06

**Authors:** Michael G. Weller

**Affiliations:** 1 Division 1.5 - Protein Analysis, Federal Institute for Materials Research and Testing (BAM), Berlin, Germany

This article is a valuable and welcome contribution to the ongoing discussion of antibody quality. The suggestion of a two-tier approach is helpful to distinguish between simple descriptive data and the validation of an antibody for a specified application.

 For an updated article some minor changes should be considered:

1. Validation is a very old concept in analytical chemistry and therefore, some definitions have already emerged and many regulations and guidelines, e.g. Thompson et al. ^1^, have been put into effect. Validations of antibody-based methods should make use of these established and proven approaches and should be seen in this context.
2. In Table 1 the declaration of "Specified IgG in µg or mg" should be discussed briefly, since only in very rare cases the relevant amount has been determined properly. Either non-specific IgG (e.g. antibodies based on ascites) and IgG of a different species (such as bovine IgG) may contaminate the product. In many cases, only the protein content was determined with a semiquantitative spectrophotometric method.
3. "Comparison between wildtype and knock-out is in all cases the best validation and is not incorporated in this schedule." I want to mention that there are research antibodies against non-proteinaceous targets, which can not be validated this way. Furthermore, in the case of chemically defined antigens (e.g. peptides), the use of LC-MS/MS is perhaps the most powerful approach to validate an antibody-based method.
4. "This omission has been recognized and publishers are no longer expected to accept a paper without the catalogue numbers of the antibodies used." I do not think that a catalogue number is sufficient for this purpose. A clone number or a real antibody ID would be much better to make an antibody fully traceable ^2^.

I have read this submission. I believe that I have an appropriate level of expertise to confirm that it is of an acceptable scientific standard.

## References

[1] ThompsonMEllisonSWoodR: Harmonized guidelines for single-laboratory validation of methods of analysis (IUPAC Technical Report). *Pure and Applied Chemistry*.2002;74(5) : 10.1351/pac200274050835 10.1351/pac200274050835

[2] WellerMG: Quality Issues of Research Antibodies. *Anal Chem Insights*.2016;11: 10.4137/ACI.S31614 21-7 10.4137/ACI.S31614 27013861PMC4803150

# Peer review: Referee response for version 1

**DOI:** 10.5256/f1000research.11700.r20353

**Published:** 2017-03-01

**Authors:** Fridtjof Lund-Johansen

**Affiliations:** 1 Department of Immunology, Oslo University Hospital, Rikshospitalet, Oslo, Norway

The author provides a good overview of current issues with antibody validation. His proposal for a tiered approach to validation is well in line with suggestions from a recent workshop organized by the Global Biological Standards Institute (GBSI) ^1^ and also with published guidelines from the International Working Group on Antibody Validation (IWGAV) ^2^. My main comment relates to the choice of controls.

 The author explains that "validation always involves comparison between expressing and non-expressing cells or tissues at identical antibody dilutions". In fact, reputable antibody manufacturers rarely show negative controls in their product specification sheets. This is not surprising since there is no comprehensive and definitive source of information about the distribution of proteins in tissues, cells or subcellular compartments. There are now precise maps for the transcriptome, and some researchers argue that mRNA levels are predictive of protein abundance ^3^^,^^4^^,^^5^^,^^6^. However, published data do not provide a definitive answer to this question, so this remains a controversial issue. In my view, the author should discuss the problems associated with finding bona-fide negative controls for application-specific antibody validation.

I have read this submission. I believe that I have an appropriate level of expertise to confirm that it is of an acceptable scientific standard.

## References

[1] BakerM: Biologists plan scoring system for antibodies. *Nature*.2016; 10.1038/nature.2016.20712 10.1038/nature.2016.20712

[2] UhlenMBandrowskiACarrSEdwardsAEllenbergJLundbergERimmDLRodriguezHHiltkeTSnyderMYamamotoT: A proposal for validation of antibodies. *Nat Methods*.2016;13(10) : 10.1038/nmeth.3995 823-7 10.1038/nmeth.3995 27595404PMC10335836

[3] UhlénMFagerbergLHallströmBMLindskogCOksvoldPMardinogluASivertssonÅKampfCSjöstedtEAsplundAOlssonIEdlundKLundbergENavaniSSzigyartoCAOdebergJDjureinovicDTakanenJOHoberSAlmTEdqvistPHBerlingHTegelHMulderJRockbergJNilssonPSchwenkJMHamstenMvon FeilitzenKForsbergMPerssonLJohanssonFZwahlenMvon HeijneGNielsenJPonténF: Proteomics. Tissue-based map of the human proteome. *Science*.2015;347(6220) : 10.1126/science.1260419 1260419 10.1126/science.1260419 25613900

[4] MeléMFerreiraPGReverterFDeLucaDSMonlongJSammethMYoungTRGoldmannJMPervouchineDDSullivanTJJohnsonRSegrèAVDjebaliSNiarchouAGTEx ConsortiumWrightFALappalainenTCalvoMGetzGDermitzakisETArdlieKGGuigóR: Human genomics. The human transcriptome across tissues and individuals. *Science*.2015;348(6235) : 10.1126/science.aaa0355 660-5 10.1126/science.aaa0355 25954002PMC4547472

[5] WilhelmMSchleglJHahneHGholamiAMLieberenzMSavitskiMMZieglerEButzmannLGessulatSMarxHMathiesonTLemeerSSchnatbaumKReimerUWenschuhHMollenhauerMSlotta-HuspeninaJBoeseJHBantscheffMGerstmairAFaerberFKusterB: Mass-spectrometry-based draft of the human proteome. *Nature*.2014;509(7502) : 10.1038/nature13319 582-7 10.1038/nature13319 24870543

[6] EdforsFDanielssonFHallströmBMKällLLundbergEPonténFForsströmBUhlénM: Gene-specific correlation of RNA and protein levels in human cells and tissues. *Mol Syst Biol*.2016;12(10) :883 2795152710.15252/msb.20167144PMC5081484

# Peer review: Referee response for version 1

**DOI:** 10.5256/f1000research.11700.r20351

**Published:** 2017-02-24

**Authors:** Alison H. Banham

**Affiliations:** 1 Nuffield Division of Clinical Laboratory Sciences (NDCLS), Radcliffe Department of Medicine (RDM), John Radcliffe Hospital, University of Oxford, Oxford, UK

This article is a valuable contribution to the ongoing discussion regarding the importance of using properly validated antibodies to undertake robust and reproducible scientific research. The article distinguishes antibody testing, in which reactivity is seen using a specific technique, from validation, where specificity is observed using the appropriate positive and negative controls, and outlines the information provided on commercial product sheets. This article addresses key practical issues that can arise during production, storage, and shipping that can affect the quality of an antibody, even when the reagent has been well validated. Furthermore, the author proposes that distinguishing and identifying batch and aliquot information is adopted universally.

 An interesting point arising from reading this article is that despite the mention of using two independent antibodies to the target antigen to validate flow cytometry data (in the example using CD4), this information does not appear frequently on the performance specifications from vendors’ product sheets (Table 2), the exception being for immunoprecipitation. The lack of availability of comparative data (same experiment and biological samples) for multiple antibodies makes it difficult to identify the most effective reagent. It would be helpful if product sheets could display comparative data from suppliers having multiple antibodies to the same antigen. While researchers might still need to purchase another antibody, if they wished to compare the best from different manufacturers, having supportive data using an independent antibody significantly strengthens scientific conclusions.

 Minor comments

1. It might be useful for more inexperienced researchers to highlight in Table 2 what level of information is considered to be inadequate.
2. The statement that “Antibodies with a defined epitope/immunizing peptide are intrinsically more robust compared to antibodies raised to entire proteins because the limited size of the antigen increases the chance of reproducible characteristics” would benefit from further clarification. While this is true for polyclonals, a monoclonal antibody raised against an entire protein is as robust and reproducible as one recognising an immunizing peptide, as both will bind a single epitope.

I have read this submission. I believe that I have an appropriate level of expertise to confirm that it is of an acceptable scientific standard.
